# The Determinants of Performance in Biathlon World Cup Sprint and Individual Competitions

**DOI:** 10.3389/fspor.2022.841619

**Published:** 2022-03-29

**Authors:** Glenn Björklund, Marko S. Laaksonen

**Affiliations:** Swedish Winter Sports Research Centre, Department of Health Sciences, Mid Sweden University, Östersund, Sweden

**Keywords:** pacing, performance analysis, skiing, shooting, tactics

## Abstract

**Purpose:**

The present study aimed to determine the association of skiing speed (SS), range time (RT), and the number of missed targets (MT) with rank in sprint and individual biathlon competitions.

**Methods:**

Data were collected from the International Biathlon Union's database for 17 seasons (2002/2003–2018/2019). Furthermore, the biathletes were divided into three rank groups (G3, rank 1–3; G10, rank 4–10; and G20, rank 11–20). Multinominal regression was used to detect odds ratios associated with group rank for both sexes, separately.

**Results:**

MT was the only variable that was constantly related to G3 (OR 1.90–6.35, all *p* < 0.001) for both women and men. SS was associated with G3 in the last lap in the sprint for both sexes (OR 0.46–0.66, all *p* < 0.001) and RT for standing shooting (OR 1.04–1.14, all *p* < 0.05).

**Conclusion:**

These results show that shooting is the fundamental factor for performance in both competitions, but that SS is increasingly important for the last lap in the sprint for both sexes. Further, a fast RT in the standing shooting for women in individual and men in the sprint seems important for improving final rank.

## Introduction

Biathlon is a complex winter sport consisting of cross-country skiing and rifle shooting, where several physiological (Rundell and Bacharach, 1995; Laaksonen et al., 2020), biomechanical (Sattlecker et al., 2014; Stöggl et al., 2015; Köykkä et al., 2021), and psychophysiological factors (Laaksonen et al., 2018) affect the performance. All these factors have an impact on the final rank, which is determined by skiing time (speed), shooting accuracy, and shooting time (speed). Biathletes compete in four different individual competition types (sprint, individual, pursuit, and mass start), which differ in skiing distance, number of shooting occasions as well as the order of shooting bouts. In addition, the starting procedure differs between these competition types, i.e., sprint and individual competitions have individual starts with a 30-s start interval between biathletes whereas in pursuit and, especially in mass start, the biathletes start at the same time. Thus, the different starting procedures may have an impact on tactical and pacing components between sprint and individual competitions in comparison to pursuit and mass start. Biathlon sprint (skiing distance 7.5 and 10 km for women and men, respectively) consists of three skiing laps interspersed by two shooting occasions, one in prone and one in standing position. On the other hand, in the individual competition (skiing distance 15 and 20 km for women and men, respectively), the biathlete skis five laps and has four shooting occasions (prone, standing, prone, and standing). In sprint, each missed target results in a penalty loop of 150 m (lasting ~22–25 s), whereas in the individual competition, each missed target generates a 1-min penalty, which is added to the skiing and range times (RTs).

Earlier investigations have revealed that overall skiing speed (SS) has increased in biathlon pursuit and mass-start competitions since season 2002/2003 (Björklund et al., 2021). Moreover, there are some indications that SS also increased in sprint competitions (Laaksonen et al., 2018). However, the number of missed targets (MT) and the time spent on the shooting range (RT) have not changed in a similar fashion compared to SS (Björklund et al., 2021). In addition, the development of these variables in biathlon sprint and individual competitions is currently unknown. From another point of view, SS has been proposed, based on correlational methods, to be the major factor for performance in a sprint (Luchsinger et al., 2018; Dzhilkibaeva et al., 2019), followed by the number of MT. However, in individual competitions, the contribution from these factors for final performance is more or less even, with some differences between sexes (Luchsinger et al., 2019). Interestingly, the impact of RT on final performance seems to be minimal. Björklund et al. (2021) recently observed that the influence of SS during different skiing laps as well as MT and RT during different shooting occasions affects the final performance. This further indicates the importance of pacing in biathlon pursuit and mass-start competitions. However, the impact of SS, MT, and RT during different loops and shooting occasions has not been fully studied in biathlon sprint and individual competitions.

Therefore, the aim of the present study was first, to describe the development of SS, RT, and MT over different seasons, and second, to investigate the impact of SS, RT, and MT to final rank in biathlon sprint and individual competitions. The present study also aimed to investigate the impact of SS during different skiing loops as well as RT and MT during different shooting occasions on the final rank more in detail. Based on earlier research, we hypothesized that in biathlon sprint, SS followed by MT are the most important factors for final rank, whereas RT plays a minor role. In addition, it was hypothesized that in individual competition SS and MT have a similar impact on final rank, and again, RT plays a minor role.

## Methods

All data were obtained from the International Biathlon Union's (IBU) datacentre, which is an openly available public domain at http://www.biathlonresults.com (International Biathlon Union, 2019) and permission was granted from IBU to use the data for scientific purposes. Data were collected for biathletes ranked 1–20 in all single sprint and individual IBU World cup competitions during the 2002/2003–2018/2019 seasons (*n* = 17 seasons). The number of sprint competitions per season was nine during seasons 2002/2003, 2015/2016–2018/2019 and 10 during seasons 2003/2004–2014/2015 and 2018/2019. Similarly, the number of individual competitions was two during seasons 2018/2019, three during seasons 2002/2003–2003/2004, 2005/2006, 2007/2008, 2011/2012–2017/2018, and four during seasons 2004/2005, 2006/2007, 2008/2009–2010/2011. The number of unique starts for the sprint was as follows: women *n* = 3,245 and men *n* = 3,264, and for the individual start, women *n* = 1,041 and men *n* = 1,073. The biathletes were further categorized into three groups separately for women and men based on their final rank in each separate competition (G3, rank 1–3; G10, rank 4–10; and G20, rank 11–20). The data were then split based on the event and checked for outliers, where any results differing more than 1.5 times the interquartile range from the mean were removed from the dataset. Thus, missing values, e.g., due to lost sensor connection or timing transponder were excluded.

### Data and Statistical Analysis

All data were pre-checked for normality using the Kolmogorov–Smirnov test. SS, RT, and MT did not conform to normal distribution. Consequently, a multi-nominal regression was used to investigate the association between SS, RT, and MT to group rank (G3, G10, and G20). For more exact comparisons, skiing time was converted to SS due to variations in the distance for the skiing tracks used by IBU. The SS for each lap was calculated as the distance of each lap divided by the skiing time for the lap, and similarly, the total SS was calculated as the total skiing distance for competition divided by the total skiing time. The analyses were performed using separate models for sprint and individual starts for women and men separately. The reference group, i.e., the base category that all other groups are related to, was set to G3 for both types of events. Comparisons between SS within sprint and individual events for women and men were analyzed using a Friedman's test to compare the laps (3 and 5 per event for SS), and RT and MT per shooting occasion for the individual competition (4 occasions per event). A Durbin Conover test was applied if Friedman's test was significant to make pairwise comparisons. A Wilcoxon signed ranked test was used to compare RT and MT in the sprint event (2 occasions per event). A point biserial correlation (*r*_*pb*_) coefficient was used as the effect size. Interpretive benchmarks were small *r*_*pb*_ < 0.10, medium *r*_*pb*_ = > 0.11– < 0.36, and large *r*_*pb*_ > 0.37 (McGrath and Meyer, 2006). Kruskal–Wallis non-parametric test was used to compare the seasons for SS (2003–2019) and RT (2012 2019) with an epsilon squared (ε^2^) for the determination of the effect size. The measurement of RT became standardized from season 2011/2012, and therefore, the use of data before that season was not reliable. Furthermore, a Dwass-Steel-Critchlow-Flinger test was applied for pairwise comparisons if there was a global significance for the Kruskal–Wallis test. For comparisons between groups related to the number of MT, a χ^2^ test of independence was applied. A Cramer's *V* (*V*) was used for effect size. MT are presented as numbers and modes. Statistical analyses were performed using *jamovi* (The Jamovi Project, 2020) and SPSS statistical package version 27 (SPSS Inc. Chicago, IL; the normality check) with data presented as odds ratios (OR) with confidence intervals (95% Cl), median (interquartile range [IQR]), or mean values, where appropriate. The α was set *a priori* to <0.05.

## Results

### Skiing Speed

**Figure 1:**
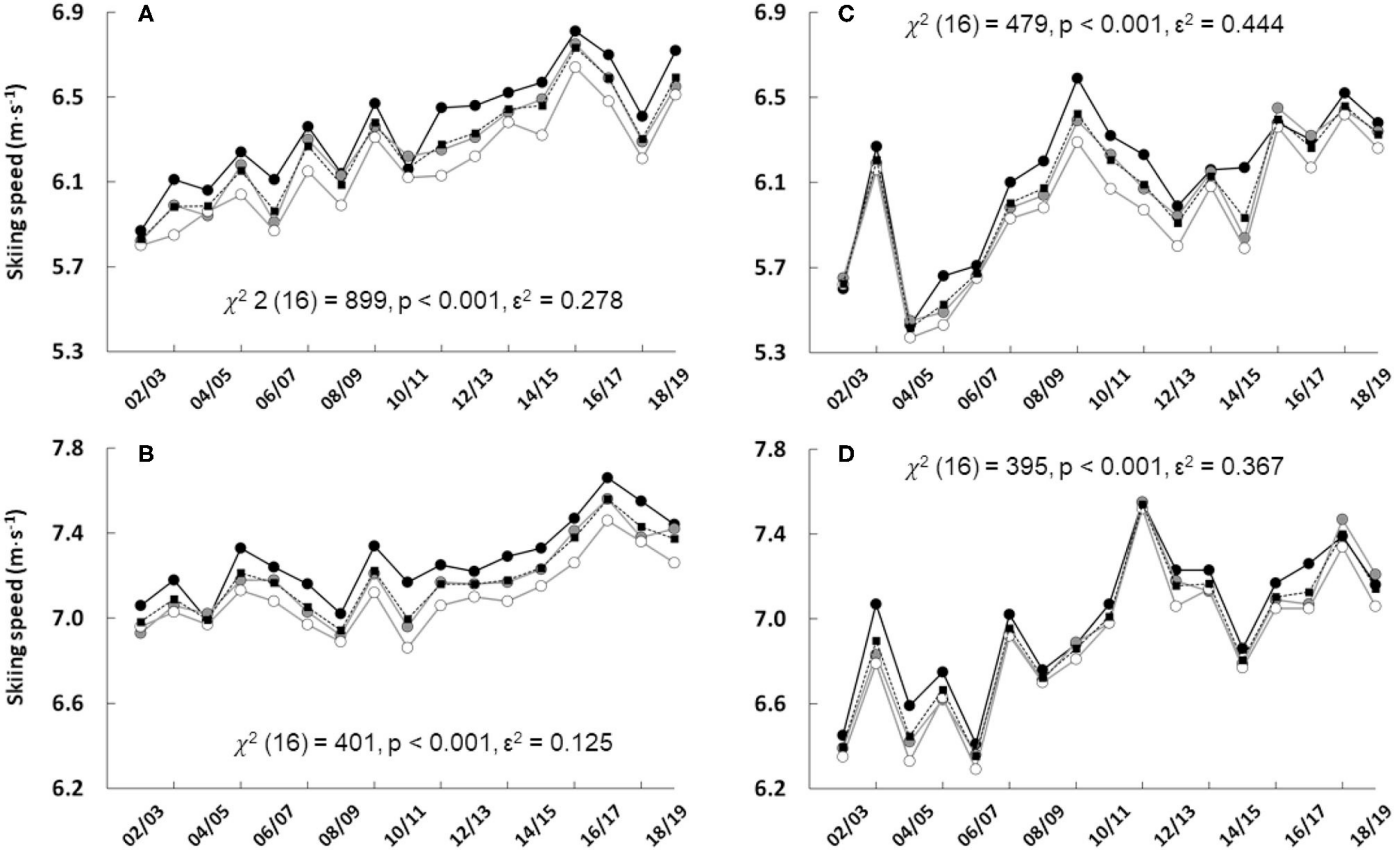
The skiing speed in sprint and individual competitions for women [**(A)** and **(C)**, respectively] and men [**(B)** and **(D)**, respectively] including the overall (black dashes line, black squares) and groups of ranks 1–3 (black solid line, black filled circles), ranks 4–10 (gray solid line, gray filled circles), and ranks 11–20 (gray solid line, white filled circles) during seasons from 2002/2003 to 2018/2019. Each data point represents the median of all competitions during each season for each group. Sprint **(A,B)** and individual **(C,D)** include 9–10 and 3–4 competitions during each season, respectively, except season 2018/2019 having only 2 individual competitions.

Over the 17 seasons of this analysis, for both sprint and individual competitions, SS increased from 2002/2003 to 2018/2019 (p < 0.001) for both women and men. There was a substantial year-to-year variability, as shown in Figures 1A–D.

**Figure 2:**
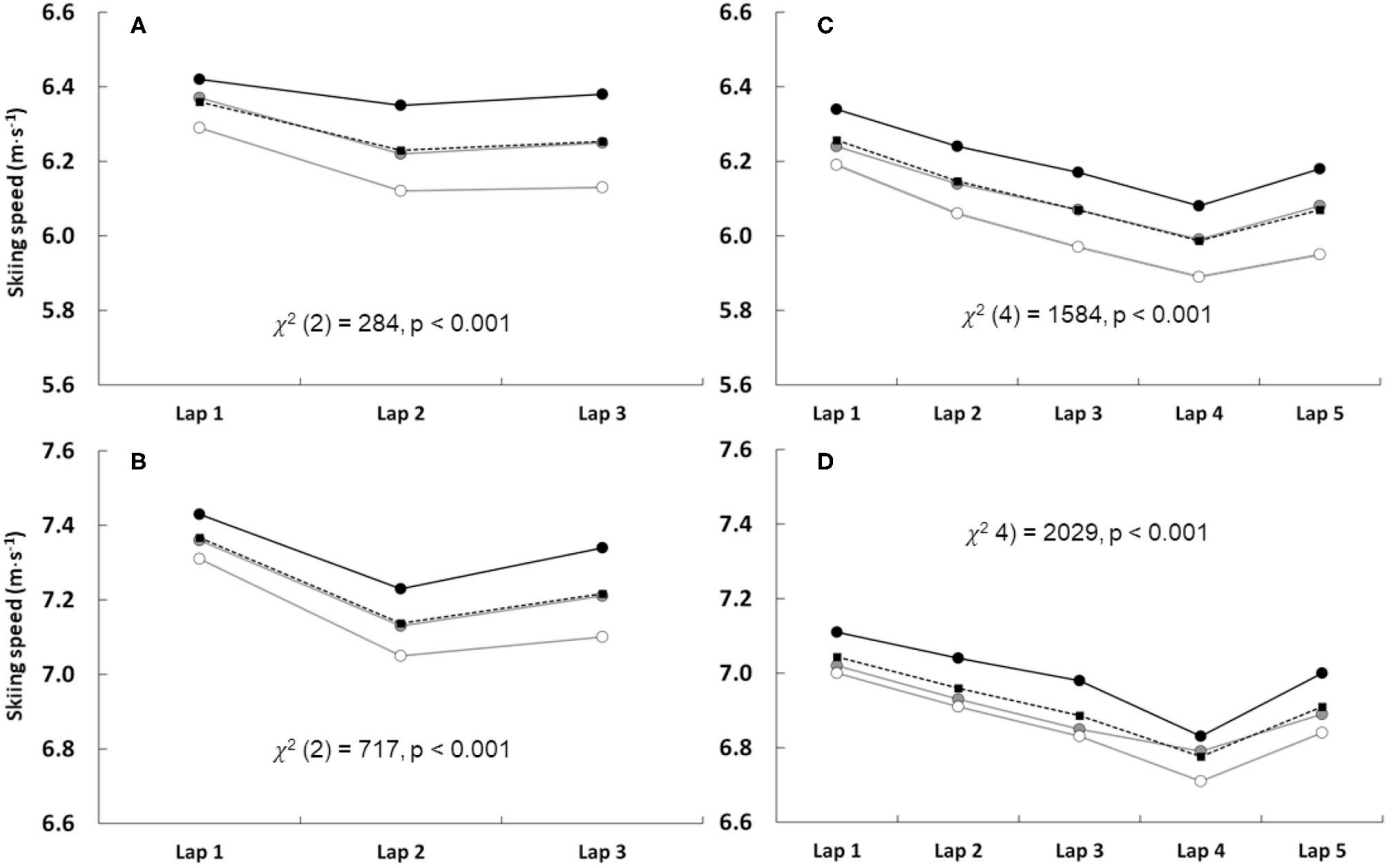
The skiing speed during different laps in sprint and individual competitions for women [**(A)** and **(C)**, respectively] and men [**(B)** and **(D)**, respectively], including the overall (black dashed line, black squares) and groups of ranks 1–3 (black solid line, black filled circles), ranks 4–10 (gray solid line, gray filled circles,) and ranks 11–20 (gray solid line, white filled circles) during seasons from 2002/2003 to 2018/2019. Each data point represents the median of all competitions during each season for each group. Sprint **(A,B)** and individual **(C,D)** include 9–10 and 3–4 competitions during each season, respectively, except season 2018/2019 having only 2 individual competitions.

In the sprint, SS changed between laps, with the first lap being the fastest for both women and men [women = 6.34 (5.87–6.72) m·s^−1^, Figure 2A; men = 7.35 (7.03–7.63) m·s^−1^, Figure 2B] and the second lap the slowest [women = 6.19 (5.85–6.51) m·s^−1^; men = 7.11 (6.81–7.35) m·s^−1^]. In both women's and men's sprint races, G3 was strongly associated with faster SS during the second lap compared to G20, and during the last lap compared to both G10 and G20 (Table 1).

**Table 1:** Multi-nominal logistic regression for skiing speed in women's and men's sprint and individual competitions in IBU WC 2002/2003–2018/2019.

**Sprint-start**	**Women**			**Men**		
**Group**	**OR**	**95% CI**	***p*** **-value**	**OR**	**95% CI**	***p*** **-value**
G10–G3						
Lap 1	1.24	0.87–1.78	=0.241	1.07	0.74–1.56	=0.716
Lap 2	0.66	0.39–1.11	=0.114	0.70	0.43–1.14	=0.153
Lap 3	0.66	0.48–0.91	=0.010	0.65	0.47–0.91	=0.012
G20–G3						
Lap 1	1.37	0.97–1.93	=0.079	1.37	0.96–1.96	=0.087
Lap 2	0.48	0.29–0.80	=0.005	0.48	0.30–0.77	=0.002
Lap 3	0.49	0.36–0.66	<0.001	0.46	0.34–0.64	<0.001
**Individual-start**	**Women**			**Men**		
G10–G3						
Lap 1	1.74	0.66–4.57	=0.263	1.22	0.48–3.08	=0.674
Lap 2	1.19	0.08–16.71	=0.900	2.47	0.25–24.15	=0.438
Lap 3	0.73	0.03–20.07	=0.849	0.13	0.01–1.95	=0.140
Lap 4	0.45	0.03–6.32	=0.552	3.36	0.37–30.59	=0.282
Lap 5	0.84	0.41–1.71	=0.631	0.59	0.28–1.22	=0.151
G20–G3						
Lap 1	3.36	1.32–8.54	=0.011	1.42	0.58–3.46	=0.443
Lap 2	1.74	0.14–22.28	=0.672	6.74	0.75–60.65	=0.089
Lap 3	0.13	0.01–3.14	=0.206	0.24	0.02–3.19	=0.278
Lap 4	0.77	0.06–9.91	=0.838	0.60	0.07–4.96	=0.637
Lap 5	0.55	0.28–1.09	=0.089	0.44	0.22–0.87	=0.019

*Lap: G3, rank 1–3; G10, rank 4–10; G20, rank 11–20. Data are reported as odds ratio (OR) with a 95% confidence interval (95% Cl) and p-values*.

In the individual start, SS resembled the same pattern for both women and men with the first lap being the fastest [women = 6.24 (5.81–6.56) m·s^−1^, Figure 2C; men = 7.03 (6.72–7.54) m·s^−1^; Figure 2D] and the fourth lap being the slowest [women = 5.95 (5.61–6.25) m·s^−1^; men = 6.75 (6.39–7.05) m·s^−1^]. In the women's individual competition, a faster SS was only associated with rank during the first lap (G20 vs. G3, *p* = 0.011). There were no associations between SS and rank in all the other laps (Table 1). In the men's individual competition, the last lap was associated with G3 in comparison to G20 (*p* = 0.019; Table 1).

### Range Time

**Figure 3:**
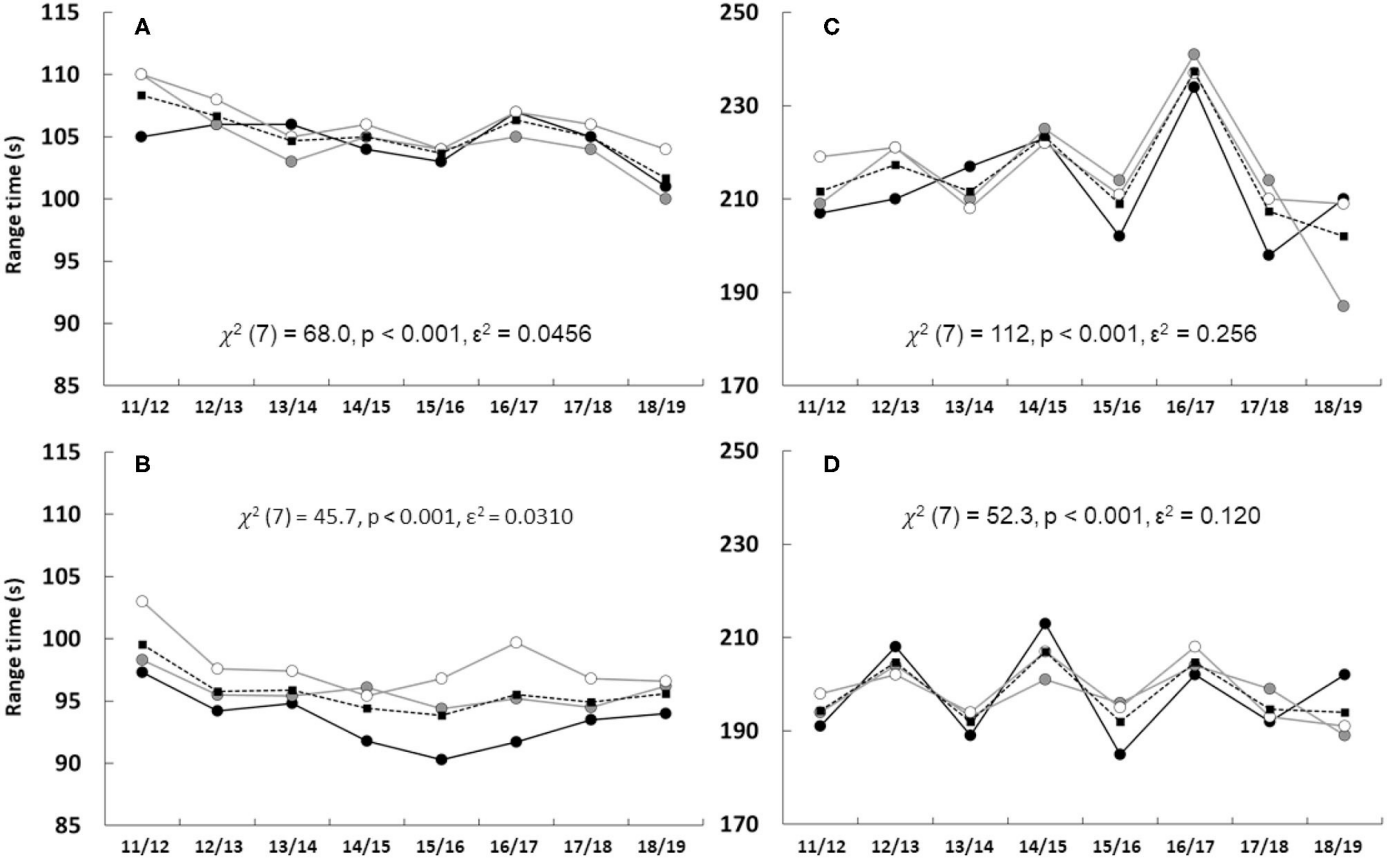
The total range time in sprint and individual competitions for women [**(A)** and **(C)**, respectively] and men [**(B)** and **(D)**, respectively] including the overall (black dashed line, black squares) and groups of ranks 1–3 (black solid line, black filled circles), ranks 4–10 (gray solid line, gray filled circles) and ranks 11–20 (gray solid line, white filled circles) during seasons from 2002/2003 to 2018/2019. Each data point represents the median of all competitions during each season for each group. Sprint **(A,B)** and individual **(C,D)** include 9–10 and 3–4 competitions during each season, respectively, except season 2018/2019 having only 2 individual competitions.

As can be seen in Figure 3, RT varied substantially between seasons for both women (Figures 3A,C) and men (Figures 3B,D). Consequently, there were no trends for an overall faster RT for either women or men from 2011/2012 to 2018/2019 in sprint or individual competitions.

For the sprint event, RT was fastest during the standing shooting [women = 51.6 (48.1–55.2) s; men = 46.4 (43.4–49.6) s] and slowest during the prone shooting [women = 53.7 (49.9–57.4) s; men = 49.7 (46.6–53.1) s] for both women and men [*z* = 14.8, *p* < 0.001, *r*_*pb*_ = 0.445 and *z* = 26.0, p < 0.001, *r*_*pb*_ = 0.686, respectively]. A fast RT during standing shooting in the women's sprint was associated with G3 compared to G20 (*p* = 0.020; Table 2). Similarly, in the men's sprint competition, a fast RT during standing shooting was associated with rank (G3 vs. G10, *p* < 0.001; G3 vs. G20, *p* < 0.001; Table 2).

**Table 2:** Multi-nominal logistic regression for range time in women's and men's sprint and individual competitions in IBU WC 2002/2003–2018/2019.

**Sprint-start**	**Women**			**Men**		
**Group**	**OR**	**95% CI**	***p*** **-value**	**OR**	**95% CI**	***p*** **-value**
G10–G3						
RT 1	0.98	0.94–1.01	=0.220	0.96	0.92–1.00	=0.054
RT 2	1.02	0.99–1.06	=0.246	1.09	1.04–1.14	<0.001
G20–G3						
RT 1	1.00	0.97–1.04	=0.983	0.975	0.94–1.01	=0.182
RT 2	1.04	1.01–1.08	=0.020	1.136	1.09–1.19	<0.001
**Individual-start**						
G10–G3						
RT 1	0.93	0.85–1.02	=0.145	1.08	0.98–1.19	=0.132
RT 2	1.13	1.03–1.24	=0.009	1.03	0.93–1.13	=0.590
RT 3	1.02	0.92–1.12	=0.737	0.87	0.79–0.96	=0.007
RT 4	0.96	0.89–1.03	=0.272	1.09	1.00–1.19	=0.045
G20–G3						
RT 1	0.96	0.88–1.05	=0.373	1.04	0.95–1.15	=0.383
RT 2	1.09	1.00–1.19	=0.049	1.05	0.96–1.16	=0.290
RT 3	1.01	0.92–1.10	=0.834	0.90	0.82–0.99	=0.036
RT 4	0.99	0.92–1.06	=0.688	1.07	0.99–1.17	=0.092

*RT, range time; G3, rank 1–3; G10, rank 4–10; G20, rank 11–20. Data are reported as odds ratio (OR) with a 95% confidence interval (95% Cl) and p-values*.

In the individual event, RT changed between shooting bouts with the second occasion (first standing) being the fastest [women = 51.9 (48.5–55.6) s; men = 47.2 (44.2–50.4) s] and the third shooting occasion (second prone) being the slowest [women = 56.8 (53.2–60.6) s; men = 52.9 (50.0–56.2) s] for both women and men [$\chi_{\left(3\right)}^{2}$ = 373, *p* < 0.001, $\chi_{\left(3\right)}^{2}$ = 515, *p* < 0.001, respectively]. In the women's individual competition, a fast RT at the second shooting occasion (first standing) was associated with rank (G3 vs. G10, *p* = 0.009; G3 vs. G10, *p* = 0.049; Table 2). For the men in the individual competition, a fast RT was associated with rank during the last prone (G3 vs. G10, *p* = 0.007; G3 vs. G20, *p* = 0.036; Table 2) and standing shooting (G3 vs. G10, *p* = 0.045; Table 2).

### Shooting Profile

The total numbers of the most frequent MT during the sprint start were for women: G3 = 0–1; G10 = 0–1; G20 1–2 and for men: G3 = 0–1; G10 = 0–1; G20 1–2 (Table 3). The most frequent total numbers of MT during the individual start were for women: G3 = 0–1; G10 = 1–2; G20 2–3 and for men: G3 = 0–1; G10 = 1–2; G20 2–3 (Table 3).

**Table 3:** Frequency of missed targets out of the total number fired shots in sprint and individual competitions in IBU WC 2002/2003–2018/2019.

**Sprint-start (total number of fired shoots *n* = 32 450)**										
**Sex**	**Group**	**0**	**1**	**2**	**3**	**4**	**5**	**6**	**7**	**Total**
W	G3	**287 (59%)**	158	40	2	1	0	0	0	488
	G10	411	**490 (43%)**	194	37	4	1	0	0	1,137
	G20	351	**705 (43%)**	410	129	23	0	2	0	1,620
	Total	1,049	1,353	644	168	28	1	2	0	3,245
χ^2^ (12, *N* = 3,245 = 312, *p* < 0.001, *V* = 0.225)										
**Sprint-start (total number of fired shoots *n* = 32 640)**										
M	G3	**297 (61%)**	153	32	7	0	0	0	0	489
	G10	429	**518 (45%)**	161	38	1	1	0	0	1,148
	G20	376	**698 (43%)**	406	123	24	0	0	0	1,627
	Total	1,102	1,369	599	168	25	1	0	0	3,264
χ^2^ (10, *N* = 32,64 = 331, *p* < 0.001, *V* = 0.225)										
**Individual-start (total number of fired shoots *n* = 20 820)**										
W	G3	55	**65 (41%)**	32	5	0	0	0	0	157
	G10	40	123	**126 (35%)**	50	20	5	0	0	364
	G20	33	116	**157 (30%)**	137	55	17	4	1	520
	Total	128	304	315	192	75	22	4	1	1,041
χ^2^ (14, *N* = 1,041 = 183, *p* < 0.001, *V* = 0.297)										
**Individual-start (total number of fired shoots *n* = 21 460)**										
M	G3	48	**84 (52%)**	27	2	0	0	0	0	161
	G10	47	**157 (41%)**	124	42	5	1	0	0	376
	G20	20	122	**200 (37%)**	139	51	4	0	0	536
	Total	115	363	351	183	56	5	0	0	1,073
χ^2^ (10, *N* = 1073 = 235, *p* < 0.001, *V* = 0.331)										

*W, women; M, men; G3, rank 1–3; G10, rank 4–10; G20, rank 11–20. Bold numbers represent mode (percent) for each group and sex*.

In the women's sprint competition, the MT values (min–max) for in prone and standing, shooting, respectively, were for G3 (0–2 and 0–3), G10 (0–3 and 0–3), and G20 (0–3 and 0–5). For men, the MT values (min–max) in prone and standing shooting, respectively, were for G3 (0–2 and 0–3), G10 (0–3 and 0–4), and G20 (0–4 and 0–4).

In the individual competition for women, the MT values (min–max) in the two-prone and two-standing shooting, respectively, were for G3 (0–1, 0–1, 0–2, and 0–2), G10 (0–3, 0–2, 0–4, and 0–4), and G20 (0–4, 0–4, 0–3, and 0–3). For men in the individual competition the MT values (min-max) in the two-prone and two-standing shooting, respectively, were for G3 (0–1, 0–2, 0–2, and 0–2), G10 (0–3, 0–2, 0–3, and 0–3), and G20 (0–3, 0–3, 0–4, and 0–3).

In the sprint, both women and men showed a greater number of MT during the standing than prone shooting (*z* = 13.8, *p* < 0.001, *r*_*pb*_ = 0.330 and *z* = 12.6, *p* < 0.001, *r*_*pb*_ = 0.306, respectively). Overall, in the women's and men's individual competitions, there was a greater number of MT in the two-standing vs. the prone shootings [$\chi_{\left(3\right)}^{2}$ = 150, *p* < 0.001 and $\chi_{\left(3\right)}^{2}$ = 110, *p* < 0.001, respectively]. There were no differences in MT between the standing or the prone shooting for either women or men (*p* = 0.818 and *p* = 0.601), although there was a trend for a greater number of MT during the first vs. the second prone shooting occasion for women (women *p* = 0.055 and men *p* = 0.103). The ORs for the shooting bouts in sprint and the individual starts are presented in Table 4.

**Table 4:** Multi-nominal logistic regression for missed targets in women's and men's sprint and individual competitions in IBU WC 2002/2003–2018/2019.

**Sprint-start**	**Women**			**Men**		
**Group**	**OR**	**95% CI**	***p*** **-value**	**OR**	**95% CI**	***p*** **-value**
G10–G3						
1st prone shooting	2.03	1.60–2.58	<0.001	2.02	1.590–2.57	<0.001
1st standing shooting	1.97	1.62–2.38	<0.001	1.90	1.560–2.30	<0.001
G20–G3						
1st prone shooting	3.21	2.55–4.04	<0.001	3.15	2.490–3.97	<0.001
1st standing shooting	3.07	2.55–3.70	<0.001	3.21	2.660–3.88	<0.001
**Individual-start**	**Women**			**Men**		
G10–G3						
1st prone shooting	2.47	1.60–3.81	<0.001	2.86	1.76–4.63	<0.001
1st standing shooting	2.92	1.79–4.78	<0.001	2.36	1.48–3.77	<0.001
2nd prone shooting	2.12	1.50–3.01	<0.001	2.20	1.54–3.16	<0.001
2nd standing shooting	2.19	1.56–3.09	<0.001	2.06	1.43–2.96	<0.001
G20–G3						
1st prone shooting	3.37	2.19–5.18	<0.001	6.35	3.91–10.31	<0.001
1st standing shooting	4.08	2.51–6.65	<0.001	4.38	2.74–6.99	<0.001
2nd prone shooting	3.31	2.34–4.67	<0.001	4.14	2.88–5.97	<0.001
2nd standing shooting	3.28	2.34–4.61	<0.001	4.88	3.38–7.04	<0.001

*Shooting; G3, place 1–3; G10, place 4–10; G20, place 11–20. Data are reported as odds ratio (OR) with a 95% confidence interval (95% Cl) an p-values*.

Between seasons from 2002/2003 to 2018/2019, the number of total MT in the sprint differed for both women [$\chi_{\left(16\right)}^{2}$ = 69.2, *p* < 0.001, ε^2^ = 0.0213] and men [$\chi_{\left(16\right)}^{2}$ = 50.2, *p* < 0.001, ε^2^ = 0.0154]. There were no clear patterns in shift in MT between different time periods in the sprint competitions for women or men. In the individual event, there was an overall change in MT for women [$\chi_{\left(16\right)}^{2}$ = 104, *p* < 0.001, ε^2^ = 0.100] and men [$\chi_{\left(16\right)}^{2}$ = 53.4, *p* < 0.001, ε^2^ = 0.0498]. In 2017/2018–2018/2019, there were significantly fewer MT compared to 2002/2003–2005/2006 in the women's individual competition (*p* < 0.001). In the men's individual competition there was no clear shift between time periods for MT.

## Discussion

Partly against our hypothesis, the main finding of the present study identified that the number of MT is the fundamental variable for success within the top 20 biathletes in both sprint and individual competitions in biathlon. This pattern is recurrent for both women and men on all shooting occasions and demonstrates that podium-placed biathletes typically prevail over the top 10 and 20 athletes due to more accurate shooting. Moreover, in the sprint event, fast SS is important for both women and men and shows a strong association to a podium rank. Additionally, a fast RT seems more important for performance in the sprint for men compared with women. In the individual start, a fast RT for women in the first standing shooting influences the final rank, whereas SS has a minor impact only.

In general, the present results show a significant increase in total SS over the studied seasons. On average, SS in biathlon sprint has increased by ~4 and 2% per 5-year period for women and men, respectively. Similarly, in individual competitions, both sexes demonstrated an increased SS of ~4% per 5-year period. This finding is in-line with earlier investigations which showed increases in SS in sprint (Laaksonen et al., 2018) as well as in pursuit and mass-start competitions (Björklund et al., 2021), and is likely due to the development of skiing material as well as course preparation. Interestingly, in individual competitions, it seems that there is more variation in the development of SS in comparison to sprint. This can be partly explained by the small number of individual competitions per season (2–4) compared to sprint, with 9 or 10 per season. The variation can also be due to changes in weather conditions between seasons, which may affect the overall SS.

Variation in SS between different laps (i.e., pacing), both in sprint and individual competitions, followed a typical J-shaped curve as also has been seen earlier in biathlon (Luchsinger et al., 2019; Björklund et al., 2021). In general, the present study identified that G3 biathletes had faster SS, as shown in Figures 1, 2, but that likelihood with higher SS to be ranked in G3 was not that obvious than in an earlier study of biathlon pursuit and mass-start competitions (Björklund et al., 2021). Indeed, in sprint, a faster SS was associated with G3 only during the second (compared to G20) and third (both G10 and G20) laps, whereas in pursuit and mass-start competitions a higher SS was associated with podium rank during almost all the five laps (Björklund et al., 2021). Thus, in biathlon sprint, SS is an important factor for final rank, but other factors, such as RT and MT, may also play a more important role than previously suggested, at least in top 20 ranked biathletes. In contrast to sprint, in individual competitions, SS during different laps had very little impact on the final rank. Indeed, only the last lap for men had an association favoring G3 compared to G20. This observation supports the earlier investigation related to biathlon individual competition where penalty time (i.e., shooting accuracy) explained ~50% of the performance difference between 1–10 and 21–30 ranked biathletes (Luchsinger et al., 2019).

Range time showed a considerably larger variation between seasons for individual compared to sprint competition. It is unlikely that this variation is related to biathletes' capability in shooting. It can be speculated that this is because there are usually only three individual competitions during each season (compared to nine sprint competitions), therefore, changes, e.g., in weather conditions or different venues make RT more sensitive in this regard. However, RT was associated with the podium-placed athletes, especially in the sprint competition for the men. Previous research comparing groups from top 10 to 21–30 ranked biathletes (seasons 2011/2012–2015/2016) has shown a very low explanatory level for both sprint and individual competition for both sexes (Luchsinger et al., 2018, 2019). While the current study used top 20 athletes, it indicates that RT during the standing shooting plays an important role for the men in sprint. RT seems to be an important factor to be ahead in both G10 and G20 groups, and likely greater than the 2% explanatory factor previously shown for biathletes ranked further apart (Luchsinger et al., 2018). In the individual competition for women, a faster RT in the first shooting improves the odds to end up in G3. Interestingly, in the men's individual competition a faster RT in the second prone shooting is associated with a poorer rank. In contrast to what seems obvious as a faster RT that should be in favor of a better rank, this is in reversed order. Possibly, the biathletes ranked outside the top 10 rush the shooting pace to make up time as they might be behind due to poor shooting and slow SS. Nevertheless, the current study is in agreement with a previous analysis in individual competitions in biathlon (Luchsinger et al., 2019) that RT has a rather small impact on the final rank.

Overall, the number of MT was the central variable that was constantly associated with group ranking in both sprint and individual events. The podium-ranked group (G3) constantly displayed fewer MT in prone and standing shooting. Accordingly, in sprint, both sexes demonstrated that for both prone and standing shooting fewer MT doubles the likelihood to be placed in G3 compared to G10. In the individual competition, biathletes were almost three times more likely to be placed in G3 compared to G10, independent of sex, with fewer MT. This indicates that shooting performance is even more important in the individual race compared to the sprint competition. This is in-line with previous reports where shooting accuracy (i.e., MT) has been suggested to explain ~50% of the final rank in individual competition (Luchsinger et al., 2019), whereas in sprint, shooting accuracy explains ~30% with remaining ~60% explained by SS (Luchsinger et al., 2018; Dzhilkibaeva et al., 2019). However, the compared groups in studies by Luchsinger et al. (2018) were further apart (1–10 vs. 20–30) compared to the current study using clusters within the top 20.

Overall, there is one clear distinction in the frequency of the number of MT between sprint and the individual start. Most often the podium-placed group for both sexes in the sprint event display zero MT, while in the individual start the number of MT is most often one. For the sprint, this is in agreement with previously published data that showed that winners in the sprint event demonstrate a pattern of zero MT (Björklund, 2018). However, in both the sprint and the individual competitions, there are rare cases where biathletes placed on the podium have as many as 3–4 MT, and in those cases, it is likely due to difficult environmental conditions. Additionally, the difference between group ranks (i.e., G3, G10, and G20) in both competition types and sexes shows that there is an overlap in MT between groups. This is different compared to pursuit and mass-start events that instead show a clear pattern of one extra MT between ranked groups of G3, G10, and G20 (Björklund et al., 2021). Interestingly, it has been proposed that without taking final rank into account, the biathlon sprint has the overall lowest hit rate compared to other events (Maier et al., 2018). This is in complete contrast to another study suggesting that when adding the final rank into account, the podium group shows the lowest number of MT in all events (Björklund et al., 2021), which is supported by the recent findings. Nonetheless, in accordance with previous research, the standing shooting seems to be the most difficult of the two as it displays the greatest number of MT for both women and men (Maier et al., 2018).

One limitation of the present study is the lower number of individual competitions per season in comparison to sprint. This may partly explain the larger variation in SS and RT between seasons together with the fact that the present study did not take weather conditions into account. Also, the venues for individual competitions were likely to differ more between seasons compared to sprint (nine to 10 competitions at 10 different venues), which implies in practical terms that most of the sprint competitions were held at the same venue during each season.

## Conclusion

In all, the central performance variable between athletes within the top 20 for both women and men is to reduce the number of MT. This holds true for both the sprint and individual competitions. Furthermore, in the sprint competition, SS is important to increase the possibility to be placed in a better-ranked group, especially during the second and third lap. Moreover, to be placed in the podium group in the sprint competitions, RT is essential at the standing shooting, especially for the men. In the individual start, women seem to benefit from a faster RT during the standing shooting.

## Data Availability Statement

The raw data supporting the conclusions of this article will be made available by the authors, without undue reservation.

## Ethics Statement

Ethical review and approval was not required for the study on human participants, in accordance with the local legislation and institutional requirements.

## Author Contributions

GB and ML designed and analyzed the data, interpreted the data, wrote the first draft of the manuscript, and drafted the final manuscript and approve the final version to be published and agree to be accountable for all aspects of the work.

## Funding

This research was funded by the Mid Sweden University and Östersund City Council financial agreement (Grant No. 2018/1758).

## Conflict of Interest

The authors declare that the research was conducted in the absence of any commercial or financial relationships that could be construed as a potential conflict of interest.

## Publisher's Note

All claims expressed in this article are solely those of the authors and do not necessarily represent those of their affiliated organizations, or those of the publisher, the editors and the reviewers. Any product that may be evaluated in this article, or claim that may be made by its manufacturer, is not guaranteed or endorsed by the publisher.
